# Remodelling of Membrane Rafts Expression in Lung Cells as an Early Sign of Mechanotransduction-Signalling in Pulmonary Edema

**DOI:** 10.1155/2011/695369

**Published:** 2011-07-13

**Authors:** Paola Palestini, Laura Botto, Ilaria Rivolta, Giuseppe Miserocchi

**Affiliations:** Department of Experimental Medicine, University of Milano-Bicocca, 48 Via Cadore, 20052 Monza, Italy

## Abstract

Membrane rafts (MRs) are clusters of lipids, organized in a “quasicrystalline” liquid-order phase, organized on the cell surface and whose pattern of molecules and physicochemical properties are distinct from those of the surrounding plasma membrane. MRs may be considered an efficient and fairly rapid cell-activated mechanism to express or mask surface receptors aimed at triggering specific response pathways. This paper reports observations concerning the role of MRs in the control of lung extravascular water that ought to be kept at minimum to assure gas diffusion, supporting the hypothesis that MRs expression is a potential mechanism of sensing minor changes in the volume of extravascular water. We present the evidence that MRs expression specifically relates to signal-transduction processes evoked by mechanical stimuli arising in the interstitial lung compartment when a small increase in extravascular volume occurs. We further hypothesize that a differential expression of MRs might also reflect the damage to precise components of the extracellular matrix caused by the perturbation in water balance and thus can trigger a molecule-oriented specific matrix remodelling.

## 1. Introduction

Membrane rafts (MRs) represent specialized portions of the cell plasma membrane involved in the signalling response to incoming stimuli. In fact, MRs may be considered an efficient and fairly rapid system to express or mask surface receptors to activate specific intracellular response pathways. MRs have been described in two forms, either flat portions of plasma membrane, named lipid rafts, or flask-like of about 70 nm in diameter, named caveolae. The latter, besides representing a receptor platform, also constitutes a potential transcellular fluid carrier through transcytosis.

This paper reports observations concerning the potential role of MRs in mechano-sensitive signalling in the control of lung extravascular water, a key point in the respiratory function. Indeed, the volume of the extravascular water ought to be kept at minimum [1] in order to assure the maximum efficiency of the air-blood barrier in the gas diffusion mechanisms. In fact, we were able to describe how the expression of MRs in pulmonary cells is modified when a perturbation of extravascular lung water is caused either by saline infusion (so-called cardiogenic model of lung edema, (CE)) or exposure to hypoxia (HE) [2]. Finally, we will discuss our results considering the phenotype of animals genetically deprived of an important protein present in caveolae, namely, Cav-1.

## 2. Membrane Rafts

The pioneering work of Singer and Nicholson [4] on biological membranes predicted the existence of domains, zones where the concentration of the components differs from the surrounding membrane environment. This prediction implied the possibility that several types of domains could exist, having different patterns of component molecules, and perhaps even coexisting within the membrane. By 1974, studies on the effects of temperature on membrane behaviour led investigators to propose the presence of “clusters of lipids” [5], and by the following year data were obtained that suggested that these clusters might be “quasicrystalline” regions, namely, a liquid-order phase, surrounded by more freely dispersed and disordered lipid bilayer [6]. In the last years, several investigations, using either artificial (liposomes) or cellular plasma membranes and a variety of techniques [7–9] confirmed the existence of “quasicrystalline” regions, suggesting that organization in domains is a common feature of biological membranes. Currently, it is generally accepted that MRs exist within the plane of the membrane, where the pattern of molecules and the physicochemical properties are distinct from the environment [10, 11]. The key difference between MRs and the rest of the membrane bilayer is the lipid composition. In fact, MRs contain cholesterol and sphingolipids at concentrations up to 50% higher than rest of the membrane and the elevated sphingomyelin levels are offset by decreased levels of phosphatidylcholine [12, 13] so that the total amount of choline-containing lipids is similar in MRs and plasma membranes. As a result of tightly packed and more saturated lipids, plasma membrane regions hosting MRs are more rigid compared to the rest of the plasma membrane [12]. 

### 2.1. Caveolae and Lipid Rafts

**Figure 1:**
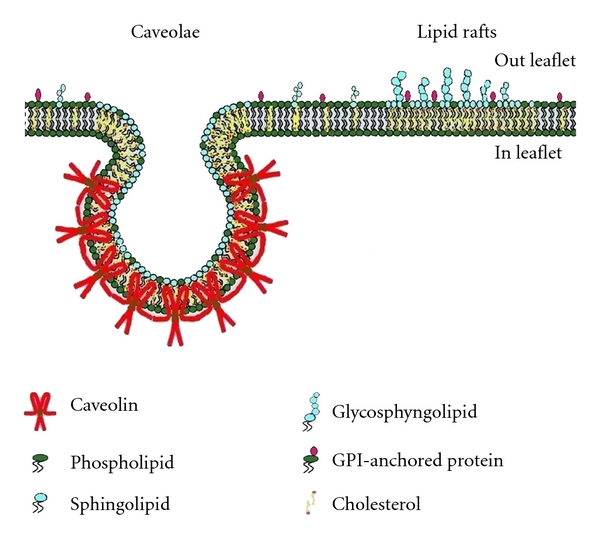
The conventionally agreed structure of caveolae and lipid rafts (adapted from Galbiati et al. [3]).

Cholesterol ought to be abundantly available for the formation of MRs; furthermore, in some domains the presence of some specific proteins is essential to form MRs. One can therefore classify protein-based membrane domains (i.e., caveolae) and lipid-based domains (i.e., lipid rafts,) [14].  Figure 1 shows the conventionally agreed structure of caveolae and lipid rafts. Caveolae are flask-shaped, about 70 nm invaginations of the plasma membrane, organized by the caveolins and the caveolin family of protein kinase C adaptors [11, 15].

Caveolins attach to the cytosolic face of the membrane via a hydrophobic hairpin loop and via a scaffolding region that interacts with cholesterol, phosphatidylserine, and PIP_2_ [16]. Removal or sequestration of cholesterol caused flask-shaped plasma membrane caveolae to flatten down and caveolin molecules to disassemble [17]. 

Lipid rafts (LRs) are small and dynamics membrane domains held together by lipid-lipid interactions. Although not necessary for raft formation, proteins could be present in these domains provided they have affinity for the domain lipid composition. LRs may differ in terms of protein and lipid composition as well as in temporal stability. 

Both caveolae and LRs are structures involved in mechanisms modulating dynamically cell function in relation to changes in plasma membrane architecture [10, 11]. Such role is proved by the finding that the action of several membrane proteins acting as receptor or second messenger generator (e.g., tyrosine kinase receptors, mono-Ras, Rap, hetero-trymeric G proteins, Src-like tyrosine kinases Lck and Fyn, protein kinase C isoenzymes, GPI-anchored proteins, and others more) was associated to expression of MRs [18–21]. This finding suggested that MRs are spatially organized on plasma membrane so as to present signalling molecules in order to promote kinetically favourable interactions for signal transduction. Conversely, these microdomains can also separate signalling molecules, to inhibit interactions in order to damp the signalling responses [22]. 

Caveolae and LRs exist predominately at the cell surface as separated structures [23], although they can sometimes associate with each other. Whenever caveolae are present on the plasma membrane, LRs are present too, conversely, LRs may exist without caveolae [24, 25]. 

Detergent insolubility is the main chemical feature of MRs. Membranes recovered as low-density fractions after cold nononic detergent extraction (i.e., Triton X-100), referred to as detergent-resistant membrane (DRM) rich in cholesterol, sphingomyelin, and glycolipid, contain MRs [26, 27].

Many subfractionation techniques based on detergent-resistant and/or low buoyant densities fractions [28–30] tend to coisolate caveolae and lipid rafts [23, 31]. Other detergents, including Lubrol WX, Lubrol PX, Brij 58, Brij 96, Brij 98, Nonidet P40, CHAPS, and octylglucoside have also been employed to prepare DRM [32, 33]. 

Unsurprisingly, the MRs composition differed to some extent as a function of the detergent used [34]. This finding may reflect either the differences in the subfractionation techniques as well as the heterogeneity of MRs, as suggested by immunoelectron [35–37] or immunofluorescence microscopy [38, 39]. 

The existence of LRs, as well as their dimensions, has been controversial owing to the difficulty of visualizing them on cell membranes. A controversy is reflected in their putative size ranging from 5 up to 200 nm, that corresponds to the resolution of optical microscopy. Electron microscopy provides the required resolution, but not in living cells. Atomic force microscopy and Forster resonance energy transfer (FRET) [40] could provide indications on LRs size, but the direct visualization of LRs, at variance with that of clustered proteins, is challenged by the rapid diffussion of the lipid.

Moreover, more recent optical microscopy methods introduced a superresolution fluorescence microscopy technique (STimulated Emission Depletion, STED), able to decrease the diffraction limit thus opening a new strategy to determine lipid diffusion [40]. The subdiffraction spot (<50 nm) created by STED is able to discriminate between freely diffusing lipids and those that are hindered. Using this technique, Eggeling demonstrated that phosphoglycerolipids, sphingolipids, and GPI-anchored proteins are transiently (about 10–20 ms) trapped in cholesterol-mediated molecular complexes dwelling within <20 nm diameter areas. 

Actually, LRs are defined as dynamics, nanoscale, sterol-sphingolipid-enriched, ordered assemblies of proteins and lipids [41]. The metastable resting state of LRs can be modified by external signals or by the initiation of membrane trafficking events, causing coalescence of LRs into larger, more stable rafts domains through specific lipid-lipid, protein-lipid, and protein-protein interactions, modulated by actin filaments.

Recent works [42] have indicated that ceramide is mainly responsible for LRs coalescence. In fact, the molecules of ceramide, released within the plasma membranes, spontaneously associate and tightly bind to other ceramide molecules inducing the formation of microceramide-enriched membranes microdomains that have however the tendency to spontaneously fuse to form ceramide-enriched macrodomains (CEM) [42]. The formation of CEM alters the biophysical membrane properties and moreover promotes the formation of highly stabilized signalling platforms with greater density of receptors. Several pathways may lead to ceramide formation although the most common one is based on the hydrolysis of sphingomyelin catalyzed by acid sphingomyelinase (ASMase). ASMase is mainly present in lysosomes and recent works [43] demonstrated that, upon stimulation, ASMase is translocated from lysosomes onto the extracellular leaflet of the cell membrane, to promote ceramide production and, consequently, CEM formation.

## 3. The Lung Model to Evaluate the Role of Membrane Rafts in Response to Alteration in Lung Water Balance

**Figure 2:**
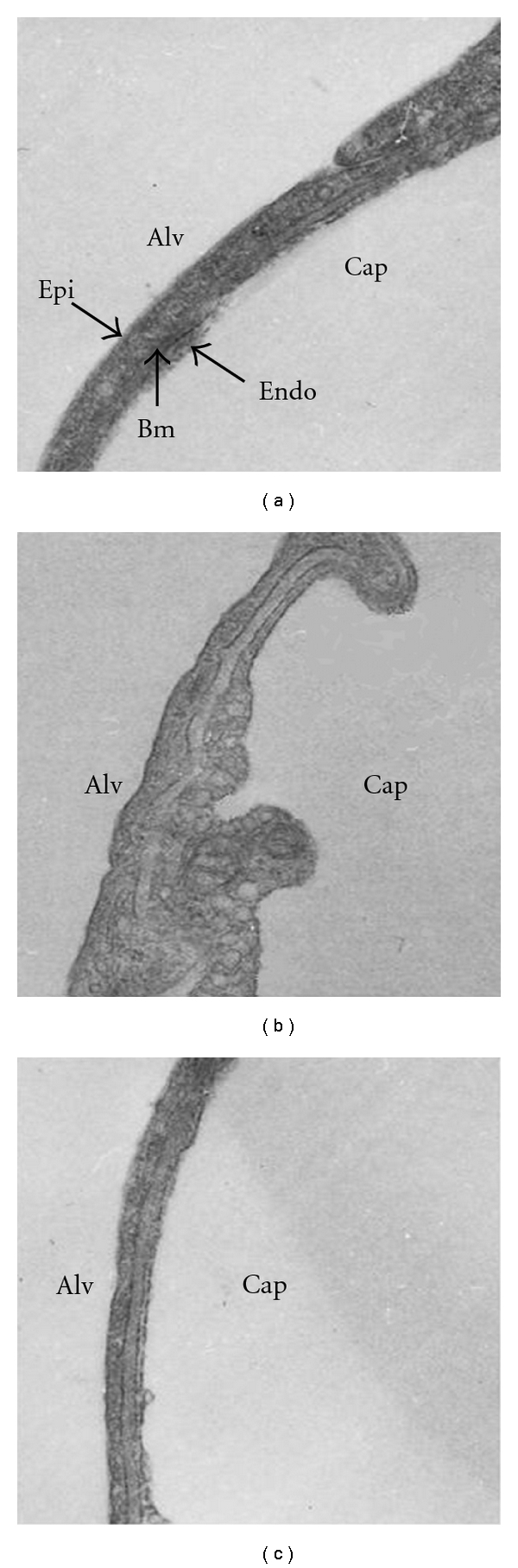
Transmission electron microscopy of the air-blood barrier in control conditions (a), after saline loading to cause cardiogenic lung edema, (CE) (b) and after hypoxia exposure to cause hypoxic lung edema, (HE) (c). From alveolar (alv) to capillary (cap) surface epithelial (Epi), basement membrane (BM) and endothelial (Endo) layer are indicated.

The lung parenchyma is composed by different cells types in particular: alveolar and endothelial (about 24 and 30%, resp.), interstitial cells including fibroblast, lymphocytes, mast cells, pericytes and plasma cells (about 36%), and macrophages (about 10%) [44, 45]. The endothelial and epithelial cells and the intervening basement membrane constitute the air-blood barrier (Figure 2(a)) that serves gas diffusion between the alveolar spaces and blood [46]. 

Alveolar epithelium is predominantly comprised of two cell types, the terminally differentiated squamous alveolar epithelial type I cell (AT-I) which constitutes approximately 93% of the alveolar epithelial surface area (estimated to be 100–120 m^2^, adult human lung) and the surfactant producing cuboidal alveolar epithelial type II cells (AT-II) comprising the remaining 7% by surface area [45]. 

Capillary endothelial cells are much smaller than AT-I, each covering an average capillary surface area that is only 27% of overall alveolar surface; the total number of capillary endothelial cells is 3-, 6-fold higher than that of AT-I, while covering the same approximate total surface area [44, 45]. 

The presence of plasmalemmal vesicles or invaginations within the AT-I cell and pulmonary capillary endothelial cell of rabbit lung has been described, for the first time, by Gil et al. [47, 48]; these workers did not distinguish between different types of vesicles but described the presence of a high numbers of noncoated vesicles or invaginations with an average diameter of 70 nm. One could make an estimate of >250 000 plasmalemmal vesicles or invaginations on the luminal membrane of an ATI cell [49]. However, in 1990 Atwal et al. [50] using electron tracer studies in goat lung, alluded to the lack of clathrin-coated pits in AT-I. The majority, but not exclusively all, of the vesicles present in the alveolar epithelium are the smaller noncoated or smooth-coated vesicle type that morphologically are recognised as caveolae. Cav-1 was localized in alveolar epithelial and pulmonary capillary surfaces of lung tissue; furthermore, by electron microscopy a greater number of caveolae was found in endothelial compared to AT-I cells, while none was found in AT-II cells [51]. 

Regarding the role of caveolae in pulmonary capillary surfaces, during recent years a growing body of experimental data has led to the general consensus that the endothelial vesicle system can mediate several processes [49, 52–54], such as transendothelial transport (transcytosis) of macromolecules [55–59] and water and modulation of vasomotion and angiogenesis through inhibition of VEGF [60] and eNOS [61, 62]. The sequestering of eNOS in caveolae is one of the major mechanisms of eNOS inhibition in endothelial cells [63, 64], and the dissociation of eNOS from caveolae is indeed required for its enzymatic activity to allow synthesis of NO [65]. Moreover, several lines of evidence now suggest that Cav-1 might play a pro-atherogenic role in endothelial cells as it is upregulated on LDL exposure while downregulation is associated with reduced uptake of oxidized LDL [66, 67]. 

In conclusion, regulation of endothelial function may be associated with MRs dissociation or endocytosis. Furthermore, MRs coalescence, mediated by ceramide as a fusogen product, to generate signalling platforms is now considered a major mechanism mediating transmembrane signalling in endothelial cells.

## 4. Water Balance in the Air-Blood Barrier

As can be appreciated from Figure 2(a), the air-blood barrier, that allows gas diffusion processes, appears extremely thin due to the paucity of water in its three compartments, namely, the endothelial and epithelial cells as well as the intervening extravascular interstitial space (the basement membrane). It is of interest to recall that the minimum volume of water in the interstitial space reflects a powerful draining action of the lymphatics in face of a very low permeability of the endothelial barrier; the resulting interstitial pressure is fairly subatmospheric, ~−10 cm H_2_O, and microvascular filtration is as low as ~1 × 10^−4^ mL/cm^2^.

In the alveolar and endothelial cells, cytoplasm volume is also kept very low, reflecting a complex balance between plasma membrane channel-mediated water fluxes and shape assumed by cells due to their strong attachments to adjacent cells and to the basement membrane. 

Fluid filtration across the capillary endothelium is defined by the revisited Starling equation:

(1) Jf=Kf[(Pc−Pi)−σ(Πc−Πi)],

where *Kf*  is the filtration coefficient, *P* and Π refer to hydraulic and colloid osmotic pressures, subscripts *c* and *i* refer to capillary and interstitial compartments, and *σ* is the protein reflection coefficient [68]. The filtration coefficient *Kf*  is equal to the product *Lp* × *S*, where *Lp* is the water hydraulic permeability and *S* is the total surface available for filtration. *Lp* mostly reflects the distribution of small pores, of the order of 5 nm, in the paracellular regions through which most of water flows. The protein reflection coefficient *σ* defines how easy it is for a the molecule to cross the endothelium based on the ratio of its radius to that of the pore. The value of *σ* varies between 0 (free passage of the protein) to 1 (full restriction of protein passage) [68]. Under physiological conditions, *Lp* is very low and *σ* is close to 1. 

The lung is by nature exposed to conditions causing an increase in microvascular filtration. This may occur through an increase in overall surface of filtration (coefficient *S*, included in *Kf*) due to capillary recruitment, normally associated with increase in cardiac output such as during exercise and/or exposure to hypoxia. Other important causes of increase in microvascular filtration are due to an increase in permeability to water (coefficient *Lp*, also included in *Kf*) and ability of proteins to cross the endothelial barrier (decrease in *σ*). These conditions are the consequence of a loss of integrity of the intercellular matrix structure, due to protease activity or ROS production (as in hypoxia exposure) [69]. 

The normal lung, at variance with other organs that can withstand relatively large increase in tissue hydration without manifest functional impairment, requires a tight control on interstitial fluid volume to guarantee respiratory gas exchange. 

At least two mechanisms are operating to control the volume of lung extravascular water.

The first mechanisms of control is based on the remarkable increase in interstitial pressure (up to about 5 cm H_2_O) when microvascular filtration increases, in face of a negligible increase in extravascular (about 5%). Such increase in pressure buffers further filtration and at the same time it increases lymphatic flow. Thus, the first mechanism of control of extravascular water is a simple negative feedback as any increase in interstitial fluid volume acts to limit a further increase. This mechanisms resides on the integrity of the interstitial matrix, mostly the proteoglycan component that provides a low compliance of the extracellular compartment [70]. 

The second mechanism depends on the reflex precapillary vasoconstriction that occurs mainly in edematous regions [71–73]. Precapillary vasoconstriction avoids an increase in capillary pressure, a factor favouring edema formation, particularly in conditions of increased cardiac output and capillary recruitment. 

In parallel with the extreme vasoconstriction in edematous regions, vasodilatation was described in regions that are still normal. Thus, in presence of in-homogeneities in regional edema formation, a complex vascular adaptation occurs in the lung to limit edema formation on one side and, on the other, to redirect blood flow to lung regions that assure gas diffusion [73]. Experimental proof has also been provided that severe edema develops when the fragmentation process exceeds a critical threshold [2] resulting in loss of rigidity of the matrix and increase in endothelial permeability.

## 5. The Mechanical Setting Triggering the Cellular Response to a Perturbation in Fluid Dynamics in the Air-Blood Barrier

 We will now discuss the potential role of MRs expression in the early signalling-transduction mechanisms triggered by abrupt change in interstitial mechanics and structure in interstitial edema. In particular, we will stress the specificity of MRs expression in relation to the typology of edema and the time course of matrix fragmentation. In doing, so we will take into consideration the time course of the remodulation of lipid moiety of plasma membrane, based on new synthesis or lipid recycling.

The marked increase in tissue pressure (from about −10 to about 5 cm H_2_O) in interstitial edema results in an increase in parenchymal stresses and forces transmitted to focal points of cell surface by cell-matrix attachments (as depicted in Figure 7). The molecules mostly involved in cell-matrix force transmission are those belonging to the proteoglycan family (PGs), namely, heparan-sulphatePGs (0.1–0.5 MDa) mostly present in the basement membrane controlling microvascular permeability and large chondroitin-sulphatePGs (>0.5 MDa) bound to hyaluronan that fills the voids of the extravascular space and provides rigidity to the interstitial matrix. Other small molecular weight PGs are involved in cell-cell and cell-matrix interactions as well as in the cytokine network [2] regulating the traffic of the molecules within the interstitial space and promoting interactions [74, 75]. 

Different time courses of PGs degradation depending on the type of edema were described [2]. In the so-called “cardiogenic” model of edema, induced by about 30% increase in plasma volume, the fragmentation process initially caused mechanical yielding of the large chondroitin-sulphatePGs of the matrix, extending subsequently to the heparan-sulphatePGs of the basement membrane. Conversely, in a “lesional” type of edema, such as exposure to hypoxia (12% O_2_), PGs fragmentation initially involved the heparan-sulphatePGs of the basement membrane [70], thus leading to an increase in microvascular permeability to water and proteins. 

We were able to correlate the differences in the time sequence of extracellular matrix disorganization to specific expression of MRs (caveolae and lipid rafts) as well as to modifications of the plasma membrane bilayer composition in lung cells [76, 77]. 

**Figure 3:**
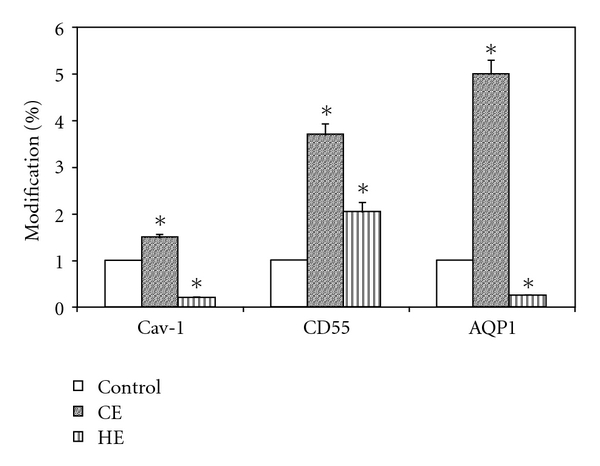
Percent changes in the expression of protein markers for caveolae (caveolin: Cav-1; aquaporin: AQP1) and lipid rafts (CD55) in cells obtained from lung tissue samples of in vivo models of cardiogenic (CE) and hypoxic (HE) lung edema.

Figure 3 shows that in cardiogenic edema there was an increase in caveolar marker (Cav-1), in line with the increased density of caveolae, as shown on TEM image in Figure 2(b); the lipid raft marker (CD55) also considerably increased. Conversely, in hypoxic lung edema a decrease in Cav-1 was found, in line with a decrease in caveolar density (Figure 2(c)), while a considerable increase in CD55 was observed. The ratio of CD55 over Cav-1 marker was 2.3 in cardiogenic edema, and it increased to 6.6 in hypoxic edema, indicating a marked increase in LRs expression, at the expense of caveolae. AQP-1, the marker of aquaporins that are expressed within caveolae, increased substantially in cardiogenic edema, but decreased in hypoxic edema, in line with the corresponding changes in Cav-1. 

**Figure 4:**
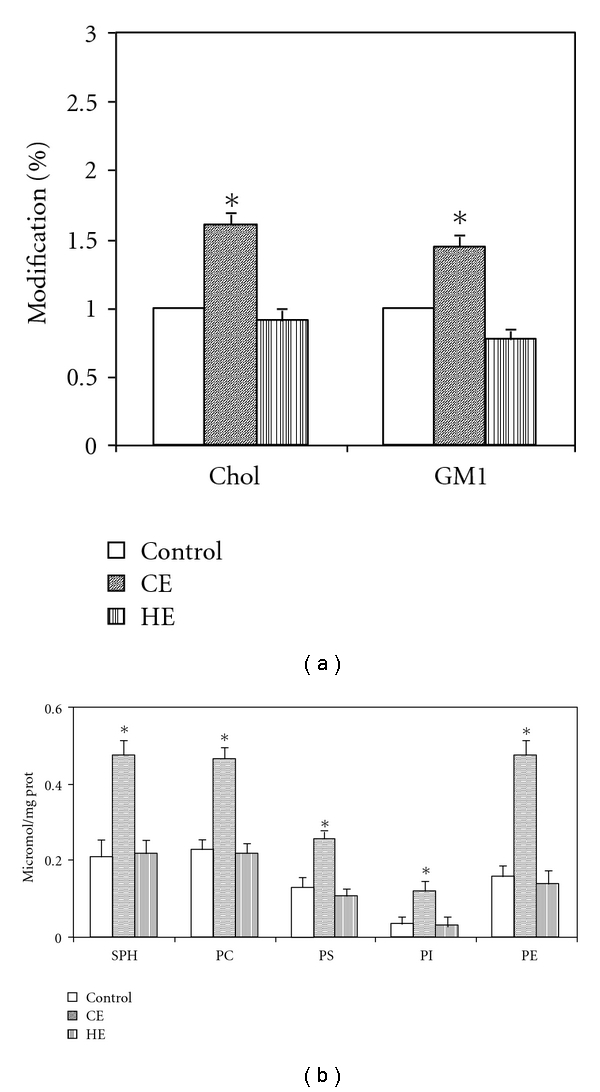
Changes in lipidic composition of lipid microdomains (caveolae and lipid rafts considered together) in cells obtained from lung tissue samples of in vivo models of cardiogenic (CE) and hypoxic (HE) lung edema. (a) Cholesterol and GM1. (b) SPH: sphingomyelin, PC: phosphatidylcholine, PS: phosphatidylserine, PI: phosphatidylinositol, PE: phosphatidyethanolamine.

Thus, differential expressions of MRs could be related to different pattern of interstitial matrix disorganisation. Regarding the lipid behaviour, the cardiogenic edema led to an increase of all principal lipid (cholesterol, GM1, and phospholipids) present in DRM that obviously include both caveolae and LRs, conversely no change was observed in hypoxic lung edema (Figure 4).

As shown in Figure 2(b), the increase in caveolar density in cardiogenic edema occurs with an increase in surface area of endothelial cells, particularly on the luminal side. This can only occur by creating new plasma membrane because these cells do not possess surface elements providing unfolding; we thus hypothesized that an increase in plasma membrane surface could occur through lipid translocation from cytoplasm to cell surface. Furthermore, although caveolae are rigid structures, the increase in cell surface with considerably irregular profile (Figure 2(b)) could be fostered by increased fluidity of the intervening plasma membrane that reflects the modifications of the posphatidylcholine/phosphatidylethanolamine and cholesterol/phospholipid ratios [77]. Cellular deformation favours vesicular formation and lipid redistribution in the plasma membrane that, in turn, may activate lipid trafficking between plasma membranes and intracellular lipid stores.

In hypoxia-induced edema, an inhibition of caveolae was found, while the quantity of lipids remained unchanged in DRM, supporting the hypothesis that these lipids may be used for caveolar disassembly and LRs formation, in line with the increase in the amount of CD55. Since no changes in total proteins markers of MRs were observed, one could suppose that these proteins are redistributed between lipid microdomains and the rest of the plasma membrane. Finally, on hypoxia exposure the flattening of the cell surface (Figure 2(c)) was accompanied by a remarkable decrease in membrane fluidity [46].

## 6. In Vitro Model

**Figure 5:**
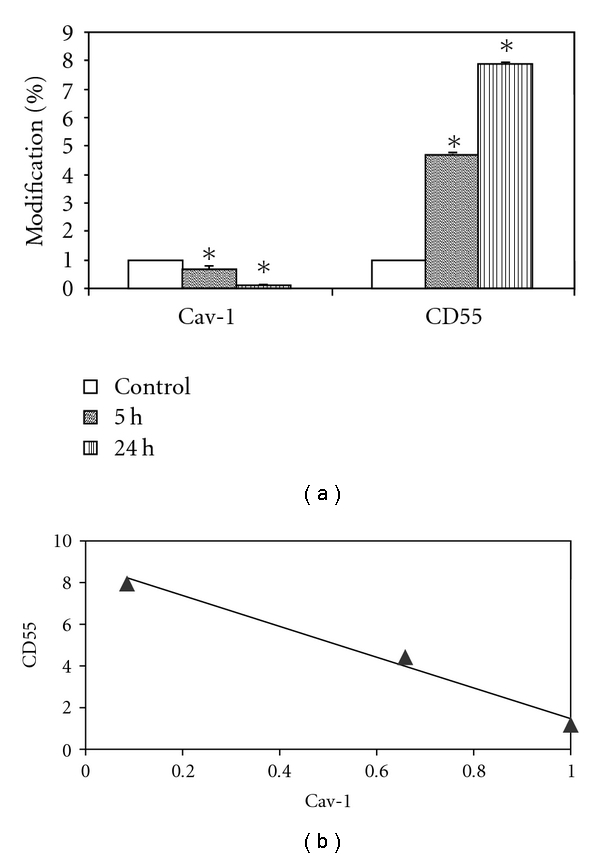
(a) Percent changes in the expression of protein markers for caveolae (caveolin: Cav-1) and lipid rafts (CD55) in cultured alveolar cells exposed to 5% oxygen for 5 and 24 h. (b) Inverse correlation between Cav-1 and CD55.

**Figure 6:**
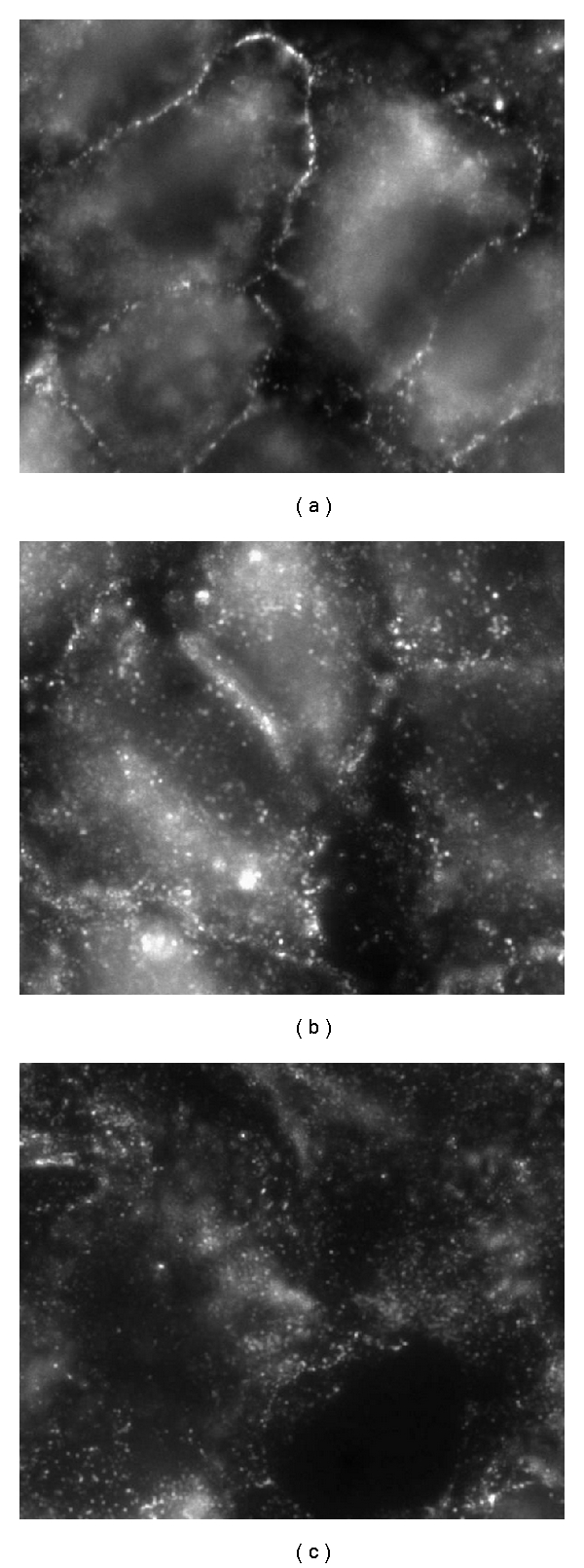
Immunofluorescence microscopy images showing the localization of Cav-1 in the plasma membrane in normoxic condition (a) and its progressive translocation within the cytoplasm after 5 and 24 h of hypoxia exposure ((b) and (c), resp.).

Botto et al. [78] using alveolar cells in culture exposed to mild hypoxia (5%, for 5 and 24 h), confirmed the inverse correlation between caveolae and lipid rafts expression observed in response to a hypoxic level similar to the in situ animal model (Figure 5), although the observed changes are smaller than those observed in the in situ model. Concerning the decrease in caveolae, no change in mRNA for Cav-1 was found, as well as in the total content of Cav-1 in total cellular membrane fraction, suggesting a shift of this protein to non-detergent-resistant fractions and/or to intracellular membrane compartments. Immunofluorescence microscopy data supported this last hypothesis as images revealed that, compared to control (Figure 6(a)), after 5 h of hypoxia exposure, Cav-1 moved away from plasma membrane surface (Figure 6(b)) towards the intracellular compartment reaching at 24 h (Figure 6(b)) a full intracellular localization. A similar process of internalization for caveolae has been described in response to physicochemical stimuli [78, 79] as, upon exposure to heat shock or hyperosmotic shock, Cav-1 was found to leave the plasma membrane moving to a region near the nuclear envelope, while in control cells, Cav-1 was not seen around the nuclear envelope. Another interesting observation is that in alveolar epithelial cells exposed to bleomycin, a chemotherapeutic agent causing lung fibrosis, Cav-1 were confined to the cytoplasm [80]. The modulation of caveolin traffic from perinuclear region to/from plasma membrane may be part of specific signalling transduction processes. 

Similarly to Cav-1, also in the case of CD55 its total content in total cellular membrane fraction was unchanged, while it increased in DRM, suggesting a shift from nonraft membrane portions. 

Sprenger et al. [81] demonstrated that LRs and caveolae proteomes are biochemically separated in endothelial cells and stressed the importance of a precise rafts-caveolae ratio to balance their interaction in controlling membrane trafficking, transduction and growth.

## 7. Consideration on the *Cav-1 (−/−)* Model

In *Cav-1 (−/−)* knockout mice, severe pathomorphological defects are observed in the lung, such as a thickening of the basement membrane in the air-blood barrier, consistent with edema formation and hypercellularity [82]. On the average, most lung regions loose the normal morphology with a marked reduction of the air/tissue volume ratio. The increased amount of extravascular water in *Cav-1 (−/−)* knockout mice can be interpreted with increased microvascular permeability due to eNOS whose action was not inhibited by caveolin [83]. 

The role of endothelial cells as mechanotransducer, is confirmed by the paper of Yu et al. [84]. In this work, using *Cav-1 (−/−)* knockout mice, the authors established that caveolae and Cav-1 are required for short- and long-term mechanotransduction in blood vessels. In fact, the endothelium lacking caveolae was unable to couple changes in blood flow with proportional vascular remodeling, suggesting that caveolae might represent an initial flow mechano-sensor directly regulated by luminal blood flow.

## 8. A Model for LM Mechanotransduction-Signalling Response

The mechanisms by which cells sense mechanical stimuli and transform the signals into intracellular biochemical signals have not been fully characterized and the most intriguing property of the caveolin/caveolae system is its involvement in mechanosensing. The two specific questions to be answered are (1) how MRs functions are regulated by mechanical and biochemical perturbations of the pericellular microenvironment and (2) what are the relationships between these stimuli and the specific changes in lipid composition of MRs and plasma membrane. 

Endothelial and epithelial cells are highly deformed in vivo being kept in a flat shape due to their strong attachments to the neighbouring cells and to the extracellular matrix. According to the “tensegrity” concept [85], their “hard-wired” cytoskeleton might keep them in a good position to respond promptly to forces/pressures applied on their surface or transmitted through the cytoskeleton [85]. In fact, the cytoskeleton has an established role in mechano-transduction being able to transmit and modulate tension within the cell via focal adhesion sites, cellular junctions, and the extracellular matrix [86, 87]. Endothelial cells are equipped with numerous receptors, such as CD31, that allow them to detect and respond to mechanical forces. Furthermore, changes in lipid composition of the bilayer [88] may represent the biochemical background to activate the signal-transduction machinery that includes MRs [89]. One can interpret the inverse correlation between caveolae and lipid rafts in response to different types of edema as a relatively fast response of the signalling system, necessary to face the cellular/extracellular microenvironment perturbation and trigger the appropriate countermeasures. 

Changes in cytoplasm volume, such as those observed in response to the two types of edema, also represent a sign of cellular activation [46]. The two edema models substantially differ in terms of proteoglycans fragments that may act as specific modulators of matrix remodelling/deposition and in cell-matrix and cell-cell interaction [90]. 

Cytoplasm volume changes are due to transmembrane ion fluxes that are part of cell transduction-signalling mechanisms [91]. In fact, the permeability of mechano-sensitive ion channels was found to vary on changing the composition of the lipid bilayer in the microenvironment surrounding the channels. Several channels have been shown to be associated with caveolae [92], such as E-Nac in AT-I cells. 

We will now discuss the potential role of ceramide, considered as a second messenger whose involvement in perturbation in lung water balance has been reported [93]. Ceramide was found to activate catabolic cytokines and matrix-metalloproteinase expression, thus promoting metabolic pathways leading to extracellular matrix remodulation and cell apoptosis [94]. 

**Figure 7:**
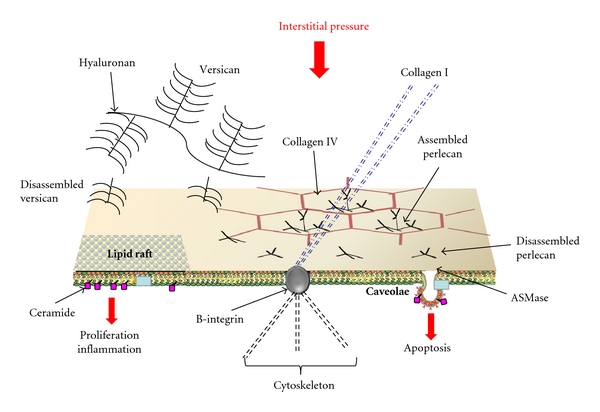
Possible model of lung cellular response to an increase in interstitial pressure caused by an increase in extravascular water not exceeding 5–10% (interstitial edema). Cell signaling is activated by mechanical stimulation through rigid links (collagen I, B-integrin, cytoskeleton), chemical/mechanical activation of the MRs by fragments of matrix and basement membrane (perlecan, versican, resp.). The activation of ASMase leads to ceramide formation that modulates different intracellular signaling pathways.

In Figure 7, we suggest a possible model of lung cellular response to an increase in interstitial pressure caused by an increase in extravascular water not exceeding 5–10% (interstitial edema) as well as to products of matrix fragmentation. The mechanical stress triggers cell signalling through rigid mechanical links (collagen I, *β*-integrin, cytoskeleton) and chemical/mechanical activation of the MRs by fragments of proteoglycans [2] from the matrix (versican) and from the basement membrane (perlecan). The activation of ASMase leads to ceramide formation that, in turn, may cause matrix remodeling and tissue repair or, conversely, matrix degradation and cell apoptosis as in degenerative lung disease. Interestingly, on clinical ground, interstitial edema ought to be considered as borderline condition between recovery or development of lung disease.

## 9. Methods

### 9.1. MRs Isolation

#### 9.1.1. In Vivo Models

The experiments were carried out on adult New Zealand rabbits [2.5 ± 0.5 (SD) kg body wt] subject to cardiogenic (CE, saline infusion type) or hypoxic (HE) lung edema. Animals were sacrificed, homogenates of the lung tissue were centrifuged, and supernatants were saved to obtain plasma membrane fractions (PM). PM were used to prepare the plasma membrane detergent resistant fraction (DRM), containing MRs [46, 76].

#### 9.1.2. In Vitro Model

A549 cells lung epithelial cells were kept in normoxia (21% O_2_) up to confluence and thereafter were exposed to hypoxia (up to 24 h 5% O_2_, 37°C). Cells kept in control and exposed to hypoxia were washed twice, harvested in PBS solution and centrifuged. The pellets were used to prepare the membrane detergent resistant fraction (DRM) [76].

#### 9.1.3. Lipid Analysis

Phospholipids, sphingomyelin, cholesterol, and glycosphyngolipid GM1 were extracted from DRMs and separated by HPTLC; detection and quantification were performed as described [46, 76].

#### 9.1.4. Protein Analysis

Cav-1, AQP1, and CD55 in DRM were determined by sequential EF-10% SDS-PAGE and Western blotting. Immunoblot bands were analysed and quantified by Kodak Image Station 2000R interfaced with a Kodak Molecular Imaging Software [46, 76].

#### 9.1.5. Statistical Analysis

Biochemical determinations were repeated three times for each animal. Biochemical results were expressed as means ± SD, averaging data from the different animals. The significance of the differences among groups was determined using one-way ANOVA and *t*-test.

#### 9.1.6. Immunofluorescence Analysis

Confluent cells were fixed with methanol at −20°C for 10 min and incubated with primary antibody Rabbit polyclonal Anti-Cav-1 (BD Italia SpA) diluted 1 : 500 in blocking buffer for 2 hrs at RT. After washing three times with HS Buffer, cells were incubated with antirabbit Alexa 488-Conjugated secondary antibody (Molecular Probes) diluted 1 : 100 in blocking buffer at RT for 1 hr. Cells were then washed three times with HS buffer and twice with LS Buffer, incubated with DAPI for 5 min and mounted with glycerol 95% in PBS. This protocol was applied in control condition and after and 24 hr of hypoxia exposure. Video confocal microscopy was performed using ViCo system on an inverted Nikon ECLIPSE TE2000E microscope with 60X objective.

#### 9.1.7. Transmission Electron Microscopy

Transmission electron microscopy was performed on lung tissue samples to carry a morphometric analysis to determine the density of caveolae in lung cells of the air-blood barrier [95].
